# Developing and Integrating Asynchronous Web-Based Cases for Discussing and Learning Clinical Reasoning: Repeated Cross-sectional Study

**DOI:** 10.2196/38427

**Published:** 2022-12-08

**Authors:** Sonny Tat, Haroon Shaukat, Pavan Zaveri, Maybelle Kou, Lenore Jarvis

**Affiliations:** 1 Division of Pediatric Emergency Medicine Benioff Children's Hospitals University of California, San Francisco San Francisco, CA United States; 2 Division of Emergency Medicine Children's National Health System Washington, DC United States; 3 Graduate Medical Education Inova Fairfax Medical Campus Fairfax, MD United States

**Keywords:** asynchronous learning, clinical reasoning, emergency medicine, pediatrics, web-based learning tool

## Abstract

**Background:**

Trainees rely on clinical experience to learn clinical reasoning in pediatric emergency medicine (PEM). Outside of clinical experience, graduate medical education provides a handful of explicit activities focused on developing skills in clinical reasoning.

**Objective:**

In this paper, we describe the development, use, and changing perceptions of a web-based asynchronous tool to facilitate clinical reasoning discussion for PEM providers.

**Methods:**

We created a case-based web-based discussion tool for PEM clinicians and fellows to post and discuss cases. We examined website analytics for site use and collected user survey data over a 3-year period to assess the use and acceptability of the tool.

**Results:**

The learning tool had more than 30,000 site visits and 172 case comments for the 55 published cases over 3 years. Self-reported engagement with the learning tool varied inversely with clinical experience in PEM. The tool was relevant to clinical practice and useful for learning PEM for most respondents. The most experienced clinicians were more likely than fellows to report posting commentary, although absolute rate of commentary was low.

**Conclusions:**

An asynchronous method of case presentation and web-based commentary may present an acceptable way to supplement clinical experience and traditional education methods for sharing clinical reasoning.

## Introduction

Clinical reasoning—how clinicians process and apply medical knowledge—is one way by which expert clinicians distinguish themselves from novices [1]. For novice medical trainees, in-person case-based experience, clinical context, and learning through observation are critical to developing clinical reasoning skills [2]. The pediatric emergency department (ED) can be an exceptional place to learn clinical reasoning skills. Patient volume and relative acuity in the pediatric ED provides real-world learning opportunities that complement traditional textbooks or didactics. However, the breadth of cases an individual trainee encounters in the pediatric ED can vary, resulting in inconsistent opportunities to hone clinical reasoning strategies. In addition, barriers of shift schedules and a busy ED can limit the sharing of clinical reasoning between providers. Finally, trainees in the ED may be only briefly observed directly by faculty, suggesting the existing apprenticeship model of learning clinical reasoning may have room for improvement [3].

Asynchronous learning—in which individuals direct their own learning at their own pace, often using web-based resources—may offer advantages uniquely suited for adult learning and emergency medicine [4,5]. A web-based asynchronous learning tool was a potentially effective way to improve knowledge in pediatric emergency medicine (PEM) [6]. An asynchronous e-learning module was associated with improved knowledge in PEM among residents, and it was similar to traditional lectures in knowledge acquisition and superior to no lectures at all [7,8]. However, most asynchronous learning interventions focus on acquiring knowledge, not sharing clinical reasoning strategies. When educational interventions do address clinical reasoning, they often focus on diagnostic reasoning while neglecting therapeutic reasoning [9].

The COVID-19 pandemic abruptly changed graduate medical education. Early studies of medical training programs across several specialties report decreased in-person clinical care experiences, missed work for COVID-19 infection or exposure, and increased remote learning [10-12]. Since the development of clinical reasoning skill is traditionally tied to in-person case-based experience, asynchronous learning approaches that focus on clinical reasoning may provide unique educational value.

To develop supplemental opportunities for clinical reasoning education that incorporates both learning through interactions with others and the unique advantages of asynchronous learning for emergency medicine, we created a web-based environment for clinicians to share case-based clinical reasoning challenges [13]. In the 8 years since its inception, we have shared over 190 user-selected cases and discussions. This paper describes the development and evaluation of this tool as well as the lessons learned from this still growing asynchronous web-based PEM case series over its initial 3-year period.

## Methods

### Procedure

We created our learning tool—called The Hot Seat—for 3 PEM fellowship programs in Virginia, Washington DC, and Maryland. The Hot Seat presents clinical cases that focus on one or multiple diagnostic or management dilemmas requiring participants to use available information to guide decision-making during various points of a patient encounter.

Cases were selected and written by PEM fellows at one of the participating programs based on a predetermined schedule. PEM fellows were advised to select cases that “raised an important diagnostic or management dilemma” and discouraged from selecting cases only because they were rare diagnoses. A brief description of the chief complaint was listed at the top of the case followed by a history and physical examination. Case presentations were modified to deidentify patients and focus on clinical challenges. Each case included several associated multiple-choice questions that intentionally had no clear right or wrong answers. The choice of case, case presentations, and associated questions aimed to frame relevant clinical reasoning dilemmas, not test specific knowledge recall. A PEM faculty advisor reviewed and edited each case and published it on a website created and customized through WordPress—a popular commercial website development tool.

Cases were published about twice per month. For each case, one PEM faculty—who was blinded to the outcomes of the case—was on the Hot Seat and tasked with explaining their clinical reasoning related to the case’s challenges, pitfalls, diagnostic pearls, disposition, or immediate management. In addition to the PEM faculty on the Hot Seat, anyone who visited the site could read the case, answer multiple-choice questions, or share their clinical reasoning strategies by posting commentary.

After a 2-week period, we published the denouement—a summary of the case discussion, including responses to multiple choice responses, clinical reasoning pearls related to the case, and the case outcome.

We developed a survey to address the specific goals of our project, specifically the acceptability and perceived utility of our novel web-based tool. We distributed a web-based survey for self-reported use and clinical relevance of the learning tool with responses on a 5-point Likert scale. We surveyed PEM practitioners at participating institutions at the end of 3 consecutive academic years (2016 to 2018) using an anonymous REDcap survey [14]. Respondents were eligible if they were members of specific institutional email lists for active PEM practitioners. Respondents self-identified their experience in PEM (fellow or PEM faculty with experience <3 years; PEM faculty with experience ≥3 years). Participants confirmed consent to participation and publication of feedback data prior to completing the survey. We counted posted comments and recorded site usage data using Google Analytics. Repeated views of a single page or multiple page views by the same user were counted as separate views.

### Ethical Considerations

This study was approved by the Children’s National Institutional Review Board (Pro00004269).

## Results

During the first 3 years of the Hot Seat, we created 55 unique cases that generated 172 comments from readers. The site had 31,417 page views. Page views varied by month, with a low of 317 page views (July 2017) to a high of 1664 page views (January 2016) and were highest around the time of publication of each new case. We sent survey invitations to about 70 providers each year and received a total of 65 completed surveys over the 3-year study period (Table 1).

**Figure 1:**
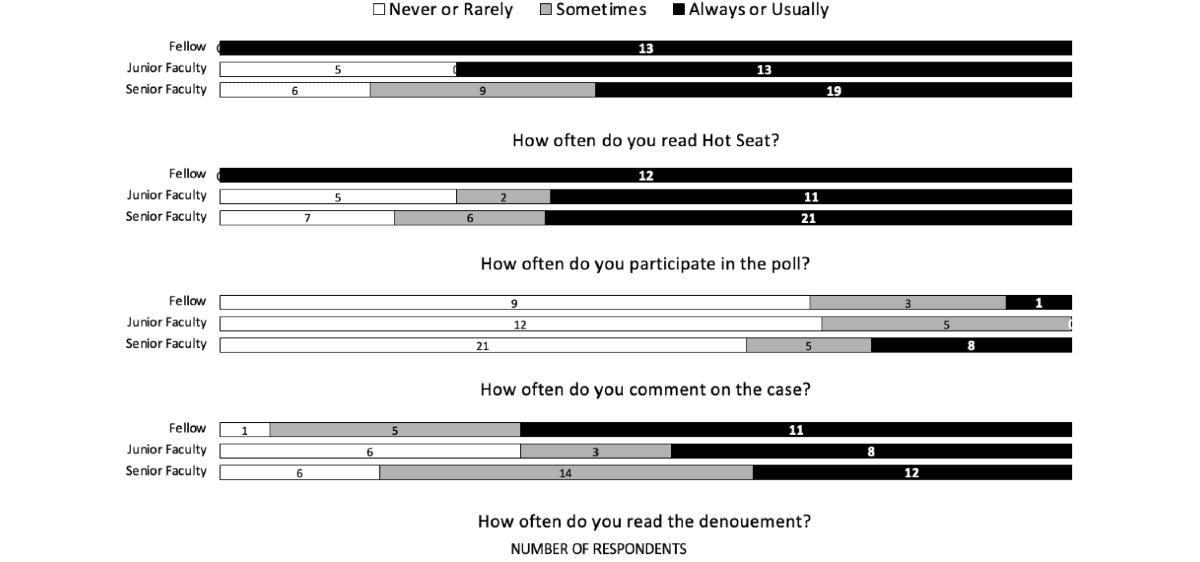
Frequency of engagement with Hot Seat by clinical experience.

The survey asked how often respondents used specific features of Hot Seat cases. The frequency of “always or usually” reading the Hot Seat cases was inversely associated with experience in PEM, with all PEM fellows “always or usually” reading the Hot Seat. For all levels of clinical experience, the proportion of respondents who “always or usually” read the denouement was smaller than the proportion who reported “always or usually” reading the case or responding to the poll. Few respondents in all groups reported commenting on cases (Figure 1).

**Figure 2:**
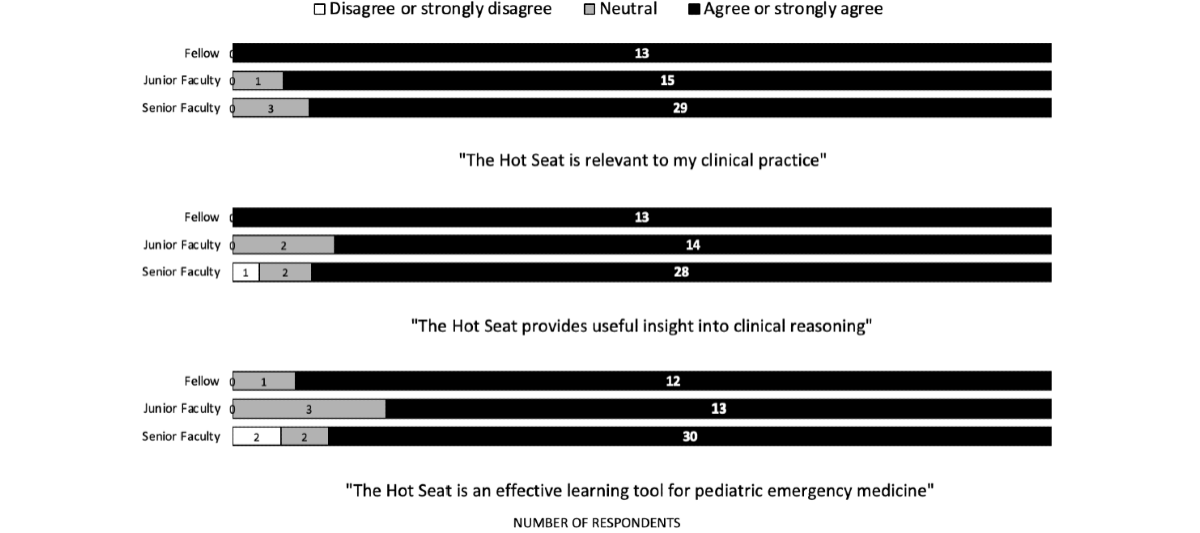
Perceived usefulness of Hot Seat by clinical experience.

All PEM fellows “agreed or strongly agreed” that the Hot Seat is relevant to their clinical practice or provides useful insight into clinical reasoning. All but one fellow also “agreed or strongly agreed” that the Hot Seat is an effective learning tool for PEM. Faculty of all levels of clinical experience generally “agreed or strongly agreed” that the Hot Seat is relevant to clinical practice, provides useful insight into clinical reasoning, and is an effective learning tool for PEM (Figure 2).

The survey included follow-up questions to better understand perceived usefulness of the Hot Seat. Of the 62 respondents who “agreed or strongly agreed” that “the Hot Seat is relevant to clinical practice,” 45 (73%) said the Hot Seat cases were similar to cases they have encountered, 58 (94%) said the Hot Seat prompted them to think about management of similar cases, and 31 (50%) said the Hot Seat cases and commentary reflected their thought processes.

Of the 64 respondents who “agreed or strongly agreed” that the Hot Seat provides “useful insight into clinical reasoning,” 55 (86%) said it was helpful to see how others approach the cases by reading the comments; 48 (75%) said the multiple-choice questions were helpful; and 46 (72%) said the cases helped them think about how they would manage similar cases in the future.

**Table 1:** Summary of survey respondents as well as Hot Seat cases and use by academic year.

Characteristics		2015-2016 (n=15)	2016-2017 (n=22)	2017-2018 (n=28)	Total (n=65)
**Clinical experience of respondents, n (%)**					
	Fellow	5 (33)	3 (17)	5 (18)	13 (20)
	Faculty <3 years	3 (20)	6 (27)	9 (32)	18 (28)
	Faculty ≥3 years	7 (47)	13 (59)	14 (50)	34 (52)
Hot seat cases, n (%)		19 (34)	18 (33)	18 (33)	55 (100)
Case comments, n (%)		57 (33)	55 (32)	60 (35)	172 (100)
Total page views, n (%)		11,632 (37)	9436 (30)	10,349 (32)	31,417 (100)
Time on page (seconds), mean (SD)		171	176	148	N/A^a^

^a^N/A: not applicable.

## Discussion

### Principal Findings

Interest and engagement in web-based medical educational resources have grown in recent years [15,16]. In comparison to many available web-based tools, the Hot Seat uniquely focuses on case discussion and clinical reasoning—important areas of medical education for which designing teaching initiatives can be challenging. In our experience, we found an engaged target audience with a range of clinical experience who reported useful insights into clinical reasoning and value in reading commentary, yet a reluctance to personally write commentary.

The proportion of respondents who engaged in the Hot Seat was high in all groups—with the PEM fellows reporting the highest share of engagement. Engaged PEM fellows were expected, since they create the cases and are presumably invested in their learning as trainees. Weekly PEM fellows’ conferences included designated time to discuss the current Hot Seat case, adding more impetus for fellows to engage. However, even among the senior clinicians, few respondents reported “rarely or never” engaging, suggesting that a web-based tool may be acceptable to a broader range of experience levels rather than just trainees.

Most respondents found value in reading the case comments and seeing how others would approach the cases. Despite the reported value of reading comments, most respondents rarely posted comments themselves. Reading, but not posting is a common social media behavior and consistent across learning platforms, where most people consume content, and a small proportion creates the majority of the content [17].

Most respondents found that comments are useful for learning, yet only about half of respondents said that the comments reflected their own thought processes. Sharing disagreements in reasoning is a feature of the Hot Seat that is distinct from many asynchronous educational approaches. Strategies to increase the sharing of reasoning and promote discussions among users may be an area of focus for future projects.

Although a small number of respondents posted comments to cases, the most experienced PEM faculty represented the group with the largest proportion of “usually or always” commenting on the cases. Experienced faculty contributing a high proportion of content is consistent with prior data on social media use in medical education [18]. Since experienced clinicians play an important role in sharing experience and clinical reasoning, future work should find ways to amplify engagement of these experienced clinical voices.

### Limitations

Accessing a case discussion is not equivalent to learning clinical reasoning. Therefore, although our site usage data provide a broad picture of readership, it does not necessarily reflect educational engagement. Our survey questions sought to address this limitation, but relatively low response rates among faculty and the self-reported nature of survey results limited our conclusions on how individuals use the Hot Seat. We created our survey to explore the goals of our study and used common questionnaire development practices. However, we did not validate the survey, which may limit the interpretation of survey results. Response bias may skew survey results toward the positive and may not be applicable to a broader audience. Finally, commentary analysis was quantitative rather than qualitative. A more complete understanding of comment quality and relevance may be a useful next step.

### Practical Lessons Learned

The Hot Seat’s blog format has advantages while also presenting challenges. The biggest advantage is the relatively low barrier to entry. WordPress has an accessible drag and drop interface, requires minimal prior coding experience, has a robust user community and associated support forums, and generally creates an affordable website format familiar to most people. On the other hand, a blog is not an ideal format to simulate a multilayered case discussion. Formatting and posting cases as well as creating the denouement require frequent, active inputs by a centralized group of people. Case commentary is typically individual statements rather than active discussions. Analytics are basic and cannot associate learning behavior to individuals, making educational assessments challenging. A future platform for clinical reasoning education should be customizable and modern. Features might include automations for creating and posting the cases, an interface conducive to discussions, and reliable learner analytics.

### Conclusions

Since we reviewed the initial 55 cases and learner data for this study, we have published over 140 additional cases. Case authors, learners, and cases have changed over time, making traditional pre- and postintervention comparisons challenging. Yet the longevity, variety, and evolving nature of our project demonstrates that clinical reasoning scenarios continually present themselves, and sharing them via an asynchronous web-based site may be an acceptable and useful approach to facilitating a clinical reasoning discussion.

## Abbreviations

ED: emergency department
PEM: pediatric emergency medicine

## References

[1] Bowen JL (2006). Educational strategies to promote clinical diagnostic reasoning. N Engl J Med.

[2] Kassirer JP (2010). Teaching clinical reasoning: case-based and coached. Acad Med.

[3] Chisholm CD, Whenmouth LF, Daly EA, Cordell WH, Giles BK, Brizendine EJ (2004). An evaluation of emergency medicine resident interaction time with faculty in different teaching venues. Acad Emerg Med.

[4] Sadosty AT, Goyal DG, Gene Hern H, Kilian BJ, Beeson MS (2009). Alternatives to the conference status quo: summary recommendations from the 2008 CORD Academic Assembly Conference Alternatives workgroup. Acad Emerg Med.

[5] Cloutier R, Walthall J, Mull C, Nypaver M, Baren J (2010). Best educational practices in pediatric emergency medicine during emergency medicine residency training: guiding principles and expert recommendations. Acad Emerg Med.

[6] Burnette K, Ramundo M, Stevenson M, Beeson MS (2009). Evaluation of a web-based asynchronous pediatric emergency medicine learning tool for residents and medical students. Acad Emerg Med.

[7] Chang TP, Pham PK, Sobolewski B, Doughty CB, Jamal N, Kwan KY, Little K, Brenkert TE, Mathison DJ (2014). Pediatric emergency medicine asynchronous e-learning: a multicenter randomized controlled Solomon four-group study. Acad Emerg Med.

[8] Pourmand A, Lucas R, Nouraie M (2013). Asynchronous web-based learning, a practical method to enhance teaching in emergency medicine. Telemed J E Health.

[9] Gruppen LD (2017). Clinical reasoning: defining it, teaching it, assessing It, studying it. West J Emerg Med.

[10] Chiel L, Winthrop Z, Winn A (2020). The COVID-19 pandemic and pediatric Graduate medical education. Pediatrics.

[11] Naifeh M, Stevenson M, Abramson E, Aston C, Li STT (2021). Early impact of the COVID-19 pandemic on pediatric resident workforce. Pediatrics.

[12] Silver LJ, Kessel A, Taurassi C, Taylor M (2022). The effect of the coronavirus-2019 pandemic on pediatric critical care fellowship training. J Intensive Care Med.

[13] Hot seat. PEM academy.

[14] Harris PA, Taylor R, Thielke R, Payne J, Gonzalez N, Conde JG (2009). Research electronic data capture (REDCap)--a metadata-driven methodology and workflow process for providing translational research informatics support. J Biomed Inform.

[15] Purdy E, Thoma B, Bednarczyk J, Migneault D, Sherbino J (2015). The use of free online educational resources by Canadian emergency medicine residents and program directors. CJEM.

[16] Mallin M, Schlein S, Doctor S, Stroud S, Dawson M, Fix M (2014). A survey of the current utilization of asynchronous education among emergency medicine residents in the United States. Acad Med.

[17] Nicolai L, Schmidbauer M, Gradel M, Ferch S, Antón S, Hoppe B, Pander T, von der Borch P, Pinilla S, Fischer M, Dimitriadis K (2017). Facebook groups as a powerful and dynamic tool in medical education: mixed-method study. J Med Internet Res.

[18] Desai T, Patwardhan M, Coore H (2014). Factors that contribute to social media influence within an Internal Medicine Twitter learning community. F1000Res.

